# Differential disruption of cell cycle pathways in small cell and non-small cell lung cancer

**DOI:** 10.1038/sj.bjc.6603167

**Published:** 2006-05-16

**Authors:** B P Coe, W W Lockwood, L Girard, R Chari, C MacAulay, S Lam, A F Gazdar, J D Minna, W L Lam

**Affiliations:** 1 Department of Cancer Genetics and Developmental Biology, British Columbia Cancer Research Center, 675 West 10th Avenue, Vancouver, BC, V5Z 1L3, Canada; 2 Hamon Center for Therapeutic Oncology Research, University of Texas Southwestern Medical Center, Dallas, Texas 75390-8593, USA

**Keywords:** array CGH, gene expression analysis, cancer genome, non-small cell lung cancer, small cell lung cancer

## Abstract

Lung cancer is the leading cause of cancer-related mortality in the world, with small cell lung cancer (SCLC) and non-small cell lung cancer (NSCLC) comprising the two major cell types. Although these cell types can be distinguished readily at the histological level, knowledge of their underlying molecular differences is very limited. In this study, we compared 14 SCLC cell lines against 27 NSCLC cell lines using an integrated array comparative genomic hybridisation and gene expression profiling approach to identify subtype-specific disruptions. Using stringent criteria, we have identified 159 of the genes that are responsible for the different biology of these cell types. Sorting of these genes by their biological functions revealed the differential disruption of key components involved in cell cycle pathways. Our novel comparative combined genome and transcriptome analysis not only identified differentially altered genes, but also revealed that certain shared pathways are preferentially disrupted at different steps in these cell types. Small cell lung cancer exhibited increased expression of MRP5, activation of Wnt pathway inhibitors, and upregulation of p38 MAPK activating genes, while NSCLC showed downregulation of CDKN2A, and upregulation of MAPK9 and EGFR. This information suggests that cell cycle upregulation in SCLC and NSCLC occurs through drastically different mechanisms, highlighting the need for differential molecular target selection in the treatment of these cancers.

Lung cancer is the leading cause of cancer-related deaths worldwide (Parkin *et al*, 2005). The disease is classified into two major histological groups: small cell lung cancer (SCLC) and non-small cell lung cancer (NSCLC). Tobacco smoke is a major etiological factor, especially in SCLC. Small cell lung cancer comprises approximately 20% of all lung cancers and exhibits a neuroendocrine phenotype while NSCLC lacks these features and makes up the remaining 80% of cases. Small cell lung cancer exhibits a more aggressive phenotype that inevitably reoccurs after initial response to chemotherapy while the clinical outcome of NSCLC is often hard to determine (Zakowski, 2003; Kurup and Hanna, 2004; Stupp *et al*, 2004). Much of our current knowledge of these subtypes has been derived from a canonical set of cell lines derived from primary tumours (Phelps *et al*, 1996). These lines have been particularly crucial in the understanding of SCLC for which surgical resection is rarely performed (Rostad *et al*, 2004).

The variation in the development and progression of SCLC and NSCLC may be a result of underlying differences in genetic alteration. Although histological classification can separate these two subtypes, previous studies using conventional genome scanning techniques such as loss of heterozygosity analysis and comparative genomic hybridisation (CGH) have shown that differences and similarities in genetic aberration exist between SCLC and NSCLC (Girard *et al*, 2000; Balsara and Testa, 2002). The limited resolutions of these methods have hampered the ability to identify discrete differences in genetic alterations, which are essential to understanding the biochemical deregulation that lead to the unique phenotypes of NSCLC and SCLC. Furthermore, the lack of a well-defined progenitor cell type for SCLC has presented a major challenge in establishing specific gene expression levels (Coe *et al*, 2006).

Owing to these limitations, it has become apparent that combining genomic and gene expression data will be essential for identifying new tumour suppressors and oncogenes (Henderson *et al*, 2005; Tonon *et al*, 2005). In addition, many genomewide platforms have proved useful in defining recurrent regions of alteration in lung cancer cells (Tonon *et al*, 2005; Zhao *et al*, 2005). With the development of whole-genome tiling path array comparative genomic hybridisation (aCGH), segmental copy number changes unique to each cell type can be defined at high resolution (Ishkanian *et al*, 2004). This technology allows the fine mapping of genomic alteration boundaries to within a single bacterial artificial chromosome (BAC) clone, identifying the precise genes potentially affected by a copy number alteration (CNA). As alterations at the DNA level are the initial events in cancer development, the gene expression changes that occur as a result of these alterations will be important in tumorigenesis.

To determine novel differences in CNA between the two lung cancer cell types, we profiled the genomes of 41 lung cancer cell lines (14 SCLC and 27 NSCLC) using the whole-genome tiling path array for CGH analysis. The integration of expression data for these regions verified our findings and identified the gene expression changes associated with CNA. Furthermore, comparing expression and copy number levels between NSCLC and SCLC without the requirement for normal expression levels circumvented a significant hurdle in the analysis of SCLC. Additionally, difference-based analysis compensates for random cell culturing artefacts, allowing insight into the clinical disease. Grouping the differentially altered genes by biological function revealed cellular pathways that may drive the pathological development of these cell types. The discovery of these genes affected by phenotype-specific CNA (PSCNA) may shed light on disease mechanisms and identify novel molecular targets for therapeutics and diagnostics.

## METHODS AND MATERIALS

### DNA samples

The 41 lung cancer cell lines described were established at the National Cancer Institute (NCI-H series) and at the Hamon Center for Therapeutic Oncology Research, University of Texas Southwestern Medical Center (HCC series) except for SW-900 and SK-MES-1 (Fogh *et al*, 1977; Phelps *et al*, 1996). These cell lines have been deposited for distribution in the American Type Culture Collection (http://www.atcc.org). DNA was extracted from 27 NSCLC: 18 adenocarcinomas (H1395, H1648, H1819, H1993, H2009, H2087, H2122, H2347, HCC78, HCC193, HCC366, HCC461, HCC1195, HCC1833, HCC3255, HCC4006, HCC827 and HCC2279) and 9 squamous cell carcinomas (H157, HCC15, HCC2450, HCC95, H520, H226, SW 900, SK-MES-1 and H2170), and 14 SCLC cell lines: nine classical (H187, H378, H889, H1607, H1672, H2107, H2141, H2171, and HCC33) and five variant (H82, H289, H524, H526, and H841). The identity of all 41 cell lines were verified by fingerprinting using the Powerplex 1.2 system (Promega) which contains nine polymorphic markers.

### Tiling path array comparative genomic hybridisation

Segmental copy number status of the 41 lung cancer cell genomes were deduced in array CGH experiments using sub-megabase resolution tiling-set (SMRT) arrays. These arrays contain 97 299 elements representing 32 433 BAC-derived amplified fragment pools spotted in triplicate on two aldehyde-coated glass slides (Ishkanian *et al*, 2004; Watson *et al*, 2004). Array hybridisation was performed as previously described (Coe *et al*, 2006; Garnis *et al*, 2006). Briefly, 200–400 ng of sample and a common reference male genomic DNA (Novagen, Mississauga, ON, Canada) were separately labelled by random priming in the presence of cyanine-5 dCTP or cyanine-3 dCTP (PerkinElmer, Woodbridge, ON, Canada), respectively. Labelled sample and reference DNA probes were combined and purified using ProbeQuant Sephadex G-50 Columns (Amersham, Baie d'Urfe, PQ, Canada). The probe mixture was precipitated in a solution containing 100 *μ*g Cot-1 DNA (Invitrogen, Burlington, ON, Canada) with 0.1 × volume 3 M sodium acetate and 2.5 × volume 100% ethanol. The DNA pellet was resuspended in 45 *μ*l of hybridisation solution containing 80% DIG Easy hybridization buffer (Roche, Laval, PQ, Canada), 100 *μ*g sheared herring sperm DNA (Sigma-Aldrich, Oakville, ON, Canada), and 50 *μ*g yeast tRNA (Calbiochem, San Diego, CA, USA) and denatured at 85°C for 10 min. Repetitive sequences were blocked at 45°C for 1 h before hybridisation. Probes were then added to array slides and placed in a pre-warmed hybridisation chamber (Telechem, Sunnyvale, CA, USA). After hybridisation for ∼40 h at 45°C, arrays were washed five times for 5 min each in 0.1 × SSC, 0.1% SDS at room temperature in the dark with agitation followed by five rinses in 0.1 × SSC and dried by centrifugation.

### Imaging and data analysis

Images of the hybridised arrays were captured through cyanine-3 and cyanine-5 channels using a charge-coupled device (CCD) scanner system (Applied Precision, Issaquah, WA, USA). Images were then analysed using SoftWoRx Tracker analysis software (Applied Precision, Issaquah, WA, USA). Spot signal ratio information was mapped to genomic coordinates and median normalised. Custom software called SeeGH was used to combine replicates and visualise all data as log_2_ ratio plots in SeeGH karyograms and exclude replicate data points which exceeded a standard deviation of 0.075 (Chi *et al*, 2004). In addition, genomic imbalances were identified using aCGH-Smooth which uses a genetic local search algorithm to identify breakpoints defining segmental DNA copy number changes by using a maximum likelihood estimation to optimise breakpoint location (Jong *et al*, 2004). As previously described, the Lambda and breakpoint per chromosome settings were set to 6.75 and 100, respectively (Jong *et al*, 2004; de Leeuw *et al*, 2004). The frequency of alteration for each BAC was then individually determined for each lung cancer cell type as described previously and plotted in SeeGH Frequency Plot to visualise areas of recurrent deletion and amplification (Coe *et al*, 2006). SeeGH software packages are available upon request at: http://www.flintbox.ca/.

### Statistical analysis of array comparative genomic hybridisation alteration frequencies

Regions of differential copy number alteration between SCLC and NSCLC genomes were identified using a stringent multistep filtering process. The occurrence of copy number gain, loss, and retention at each locus was compared between SCLC and NSCLC data sets using Fishers exact test. Testing was performed using the *R* statistical computing environment on a 3 × 2 contingency table with a *P*-value threshold of 0.05. Loci for which the same cell type exhibited an increased frequency of both gain and loss when compared to the other were then excluded from these results in order to compensate for regions demonstrating higher levels of genomic instability but not true differential patterns of alteration. Finally, regions which passed the first two criteria and demonstrated alteration frequencies differing by at least 20% occurrence in either copy number loss or gain were selected for further analysis.

### Affymetrix gene expression analysis

Affymetrix HG-U133A and HG-U133B hybridisations were performed as described in Henderson *et al* (2005). RNA expression profiles were generated for 14 SCLC and 22 NSCLC cell lines, all of which are present in the array CGH data set (H187, H378, H889, H1607, H1672, H2107, H2141, H2171,H82, H289, H524, H526, H841, H1395, H157, H1648, H1819, H1993, H2009, H2087, H2122, H2347, H3255, HCC1195, HCC15, HCC1833, HCC193, HCC2279, HCC2450, HCC366, HCC4006, HCC461, HCC78, HCC827, HCC95). Absolute expression values were log-transformed and scaled to a score between 0 and 100 using MAS 5.0 (Affymetrix, Santa Clara, CA, USA), and only probe sets demonstrating a present or marginal quality score in at least 50% of samples were considered for further analysis. Gene expression data for SCLC and NSCLC were then compared using the Mann–Whitney U test to identify genes that differed in expression between the two cell types with a *P*-value of at least 0.001. The resulting gene list was then filtered to select only those genes for which the expression change matched the direction predicted by the copy number analysis.

### Real-time polymerase chain reaction

Real-time PCR validation of expression differences between NSCLC and SCLC was performed on key genes identified through combination of array CGH and Affymetrix gene expression profiling. Five micrograms of total RNA from each cell line profiled by Affymetrix microarrays was converted to cDNA using an ABI High Capacity cDNA Archive Kit (Applied Biosystems, Foster City, CA, USA). A measure of 100 ng of cDNA was used for each real-time PCR reaction. TaqMan (Applied Biosystems, Foster City, CA, USA) gene expression assays: E2F2 (Hs00231667_m1), SOX11 (Hs00846583_s1), MAP3K4 (Hs00245958_m1), HSPH1 (Hs00198379_m1), B-actin (Hs99999903_m1), 18S rRNA (Hs99999901_s1) were performed using standard TaqMan reagents and protocols on a Biorad I-cycler (Biorad, Hercules, CA, USA). The ΔΔCt method was used for expression quantification using the average of the cycle thresholds for B-actin and 18s RNA to normalize gene expression levels between samples. Expression levels were compared between NSCLC and SCLC by a Mann–Whiney U test as performed for the Affymetrix microarray data.

### Principal components analysis

The 243 Affymetrix probe sets deregulated as a result of copy number differences between SCLC and NSCLC were subjected to principal component analysis. Analysis of the samples was performed using the Statistics Toolbox (Version 5.1) of MATLAB (Version 7.1) (The MathWorks Inc., Natick, MA, USA).

## RESULTS AND DISCUSSION

### Copy number analysis of lung cancer cell genomes

**Figure 1:**
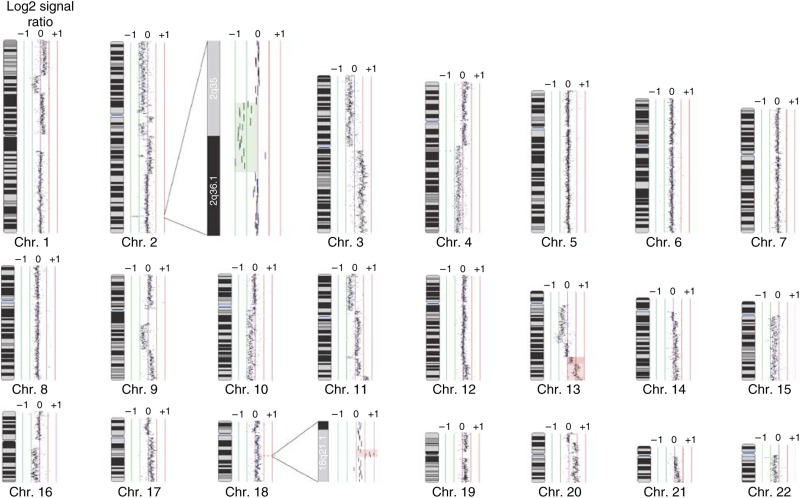
Sub-megabase resolution tiling-set (SMRT) array profile of the SCLC NCI-H1672 cells. Data is presented as a SeeGH karyogram to demonstrate the resolving power of the SMRT technology. Each BAC clone is displayed as a vertical line representing its genomic coverage. The horizontal shift of each line to the left or right of 0 represents the measured Log_2_ signal ratio from a competitive hybridisation with male genomic DNA. A decreased ratio represents a loss of copy number compared to the reference sample while an increased ratio represents and increase in copy number. Multiple levels of segmental copy number alteration as well as microalterations were readily detected (representative examples are highlighted in red and green). SeeGH karyograms for all cell lines analysed are available at http://www.bccrc.ca/cg/ArrayCGH_Group.html.

To facilitate the high-resolution search for novel genetic alterations unique to each lung cancer cell type, we analysed 14 SCLC and 27 NSCLC cell lines with the SMRT CGH array. This array allows the accurate assessment of segmental DNA copy number changes at 32 433 overlapping genomic loci in a single experiment, producing copy number maps at 100 kbp resolution across the entire sequenced human genome (Ishkanian *et al*, 2004). After co-hybridising differentially labelled sample DNA and a male genomic DNA reference, fluorescence signal intensity ratios for each array element were determined and displayed as log_2_ plots using SeeGH software. Genetic alterations were identified in all cell lines analysed. Figure 1 shows an example SeeGH karyogram for the SCLC cell line H1672. Upon visual analysis of this profile, areas of segmental gain and loss representing multiple levels of copy number change can be observed. For example, the telomeric end of chromosome arm 13q contains regions showing both single copy gain and high-level amplification (Figure 1). In addition to the multiple segmental alterations affecting the majority of chromosomes in this sample, discrete micro-amplifications and deletions are also detected such as those highlighted on chromosome arms 18q and 2q, respectively. These minute changes may have been missed by marker-based techniques and highlight the resolution of the tiling path array. Array CGH karyograms for all the cell lines are available online at http://www.bccrc.ca/cg/ArrayCGH_Group.html.

### Frequency analysis

Regions of chromosomal alteration, key to the development of tumours, will be present in multiple samples. By aligning the profiles of multiple genomes, patterns of gain and loss are revealed and minimal regions that potentially contain tumour suppressor genes and oncogenes can be identified. Thus, after generating the whole-genome tiling path array CGH profiles of the lung cancer genomes, we then proceeded to identify recurrent regions of aberration within each cell type. To do this we employed a computer algorithm, aCGH-Smooth, to aid in the automated detection of regions of chromosomal gain and loss (Jong *et al*, 2004). The frequency of alteration of each genomic locus assayed was then calculated individually for the cell types and plotted using SeeGH Frequency Plot software as previously described (Coe *et al*, 2006). The data used to generate the frequency diagrams is present in Supplementary Material. The frequency plots and a detailed description of the recurrent regions of alteration specific to these SCLC and NSCLC cell lines have been reported (Coe *et al*, 2006; Garnis *et al*, 2006).

**Figure 2:**
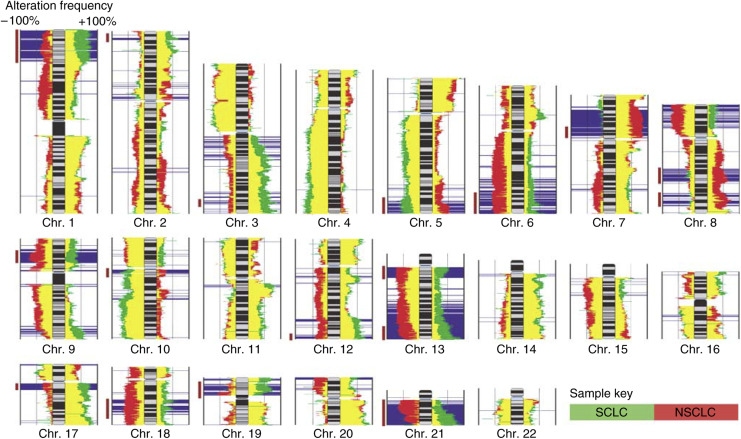
Copy number alterations in SCLC and NSCLC. Alteration frequencies for SCLC (green) and NSCLC (red) are displayed as bar plots adjacent to chromosomal ideograms. Bars extending to the right of each chromosome represent the frequency of copy number gain; conversely, bars extending to the left represent the frequency of copy number loss. Yellow regions represent overlapping portions of the SCLC and NSCLC alteration frequencies. Blue bars indicate regions demonstrating significantly different alteration frequencies. Vertical brown lines on the left of each frequency diagram indicate regions selected for further analysis.

Genetic alterations unique to each cell type may contain genes responsible for the difference in disease development and clinical behaviour. To identify these regions, we overlaid the frequency plot diagrams of the SCLC and NSCLC samples and then compared the alteration frequencies in the two groups to determine regions that were statistically different by a 3 × 2 Fishers exact test and exclusion of regions which demonstrated increased gain and loss frequency for a single cell type (Figure 2). In this figure, areas indicated in green are more frequently altered in SCLC while those in red are more frequently altered in NSCLC. The yellow represents areas of overlap between the two frequency plots. Regions shaded in blue are those determined to be differentially altered in the cell types.

### Regions of similarity

Among the regions that were not statistically different, there were some striking similarities (Figure 2). Consistent with previous reports, chromosome 3p loss was present in approximately 75% of both the NSCLC and SCLC samples (Balsara and Testa, 2002). This is consistent with previous results demonstrating that the deletion of putative tumour suppressor genes (TSGs), such as *FHIT* and *RASSF1*, contained on this chromosome arm are important genetic events in the development of lung cancers (Zabarovsky *et al*, 2002). Likewise, copy number loss of chromosome arm 4q was evident in ∼50% of samples in each cell type mirroring results observed using conventional CGH (Petersen *et al*, 1997a, 1997b)(Figure 2).

The NSCLC and SCLC cell lines also showed similar frequency of copy number gain on chromosomes arm 5p as well as at chromosome bands 7p22.3 and 11q13.1–11q14.1. Over-representation of the entire 5p arm was a recurrent event in both cell types with the telomeric end of 5p15.33 showing the greatest amount of change. This region contains the *telomerase reverse transcriptase* (*hTERT*) gene which has been implicated in cell immortalisation in numerous cancers (Tomoda *et al*, 2002; Ramirez *et al*, 2004). Gain of the 11q13.1–11q14.1 region was present in >50% of the lung cancer cell lines with the highest degree of concordance at 11q13.3 (Figure 2). *Cyclin D1*, which is involved in the inactivation of the retinoblastoma protein and progression of the cell cycle through the G1–S phase, is located at this loci (Muller *et al*, 1994). This finding supports the theory that amplification of this gene is an important event in tumorigenesis (Fu *et al*, 2004). The gain of 7p22 was particularly interesting as it was the most common copy number aberration in both cell types. The minimal common alteration within this amplified area in the SCLC cell lines contains only one gene, *MAD1L1* (validated by Coe *et al*, 2006). Although this is a checkpoint gene involved in growth inhibition, its gain has been reported in other cancers (Jin *et al*, 1999; Tsukasaki *et al*, 2001; de Leeuw *et al*, 2004). The high frequency of *MAD1L1* amplification in the NSCLC samples as well suggests that this gene may play an essential role in the development of lung cancers (Garnis *et al*, 2006).

It is noteworthy that a subset of the genomic similarities between the SCLC and NSCLC cell lines could be the result of adaptation to culturing conditions. Owing to this, the greatest insight into the biology of the clinical disease will be attainable through analysis of differences (rather than their similarities) in genomic alterations and gene regulation.

### Regions of difference

Through our analysis, numerous regions throughout the genome were determined to be differentially altered between the SCLC and NSCLC samples. This difference-based approach compensates for random cell culturing artefacts and should identify the regions most strongly linked to clinical disease. These regions ranged in size from whole chromosomes (chromosome 21) to discrete peaks, kilobases in size (3q27.1). Using our stringent, multistep criteria (Fisher's exact test followed by additional thresholding), we detected several regions that differed strongly in their alteration status between the cell types, we refer to these as phenotype-specific copy number alterations (PSCNAs). These included 1p36.33–1p34.2, 2p25.3–2p24.3, 3q26.33–3q28, 5q34–5q35.3, 6q24.2–6q27, 7p13–7p11.2, 8q21.2–8q22.3, 8q24.11–8q24.23, 9p22.3–9p21.1, 10q11.21–10q11.23, 12q24.31–12q24.33, 13q12.11–13q13.1, 13q32.2–13q34, 17q11.2, 18p11.23–18p11.21, 18q21.1–18q22.2, 19p13.2–19p12 and 21q11.2–21q22.3.

Some of these regions showed completely opposite patterns of alteration in the different cell types. 21q11.2–21q22.3 was a striking example as it is very frequently gained in SCLC but deleted in the NSCLC cases. Other regions were altered (gained or lost) in one cell type but remained almost unchanged in the other, for example the 8q21.2–8q22.3 locus that is commonly gained only in NSCLC. In addition, we observed chromosome segments altered in the same manner in both cell types, but to a greater extent in one over the other. 7p13–7p11.2 displays this characteristic as it is gained in ∼50% of the SCLC cell lines and ∼80% of the NSCLC samples.

The genes within these major regions of disparity may be responsible for the difference in disease development. However, not all genes contained in these regions will be differentially expressed as a consequence of the PSCNAs. To validate these CNAs and identify genes within these regions responsible for the different cell phenotypes, gene expression analyses were required.

### Identification of genes differentially expressed between small cell lung cancer and non-small cell lung cancer caused by phenotype-specific copy number alteration

Validation of the genomic differences identified between SCLC and NSCLC cell lines was performed by assessment of their impact at the gene expression level. This is achieved by integrating Affymetrix expression profiling data with the array CGH data presented above. Owing to the lack of a defined normal cell type for SCLC, the definition of specific over and underexpression of genes is difficult to establish. To circumvent this limitation we compared Affymetrix absolute expression values for both the NSCLC and SCLC samples to determine differential expression between the cell types.

**Figure 3:**
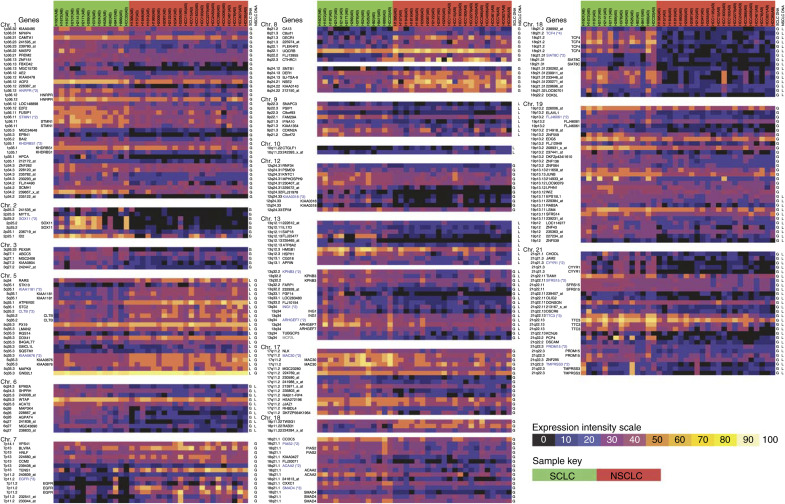
Differential expression as a result of copy number alteration. Affymetrix log-transformed absolute expression data for the 243 probe sets exhibiting strong differential expression between SCLC and NSCLC associated with copy number differences are displayed. High-level expression is indicated by white/yellow while blue/black indicates progressively lower levels of expression. The SCLC samples are indicated by green highlighting of each column, while NSCLC samples are indicated by red highlighting. Each probe set is sorted according to its chromosomal position and cell lines are sorted alphabetically, according to their cell type. Probe set with annotated gene IDs are labelled with their RefSeq name while probe sets with less reliable mapping are indicated by their probe ID alone. Average expression values were calculated for genes with multiple Affymetrix probe sets, which passed the filtering conditions. These are indicated in blue text (The number of probe sets averaged is indicated in brackets). The primary genomic alteration observed for both SCLC and NSCLC are indicated to the right of each set of expression values (G=‘gain’, L=‘loss’, no value=‘gained and lost’ or ‘no change’).

**Figure 4:**
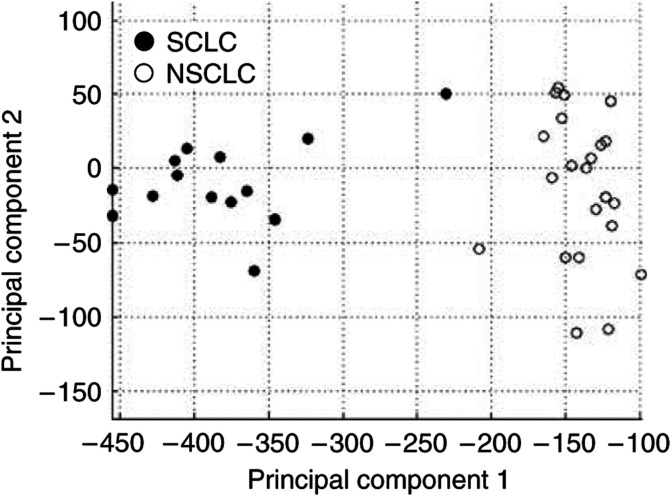
Contribution of copy number-induced gene expression differences to the SCLC and NSCLC phenotypes. Principal components analysis was performed utilising all 243 Affymetrix probe sets demonstrating expression differences as a result of copy number alterations. The SCLC samples are indicated by solid circles, while the NSCLC samples are indicated by open circles. Strong separation of the SCLC and NSCLC cell lines along principal component 1 demonstrates the contribution of these genes to the differential phenotypes.

Genes contained within the regions of peak genomic copy number difference were selected from the expression data and filtered to identify only those genes which exhibited expression differences between the two cell types presumably as a result of the copy number differences (Affymetrix gene expression data for the regions of genomic difference is available in Supplemental Material). A strict Mann–Whitney U-test *P*-value threshold of 0.001 as well as a requirement for expression differences to match the direction of copy number difference (i.e. increased expression in samples with a higher frequency of copy number gain and reduced expression in cells with a high frequency of copy number loss), identified 243 of 5185 analysed Affymetrix probe sets, corresponding to 159 unique RefSeq genes, as being differentially regulated between SCLC and NSCLC (Figure 3) (Also presented in Supplementary Material). The nature of our approach filters out genes with differential expression due to factors other than copy number such as methylation and the mutation and up/downregulation of upstream genes. As such, these 159 genes most likely represent the expression differences resulting from SCLC and NSCLC PSCNAs. This hypothesis is supported by principal components analysis, which demonstrated the strong contribution of the 159 genes to the differential phenotypes of SCLC and NSCLC (Figure 4).

Analysis of the 159 genes not only revealed several expected findings such as an increased level of EGFR expression in NSCLC, but identified novel differentially expressed genes such as *MRP5* (Amann *et al*, 2005; Ritter *et al*, 2005) which exhibited increased expression in SCLC. This gene encodes an ABC transporter known to clear various chemotherapeutics from the cytoplasm and increased expression in lung cancer has been associated with exposure to platinum drugs (Oguri *et al*, 2000). Furthermore, another study has correlated *MRP5* expression to cisplatin chemoresistant lung cancer cell lines (Weaver *et al*, 2005). This result suggests a possible mechanism of enhanced chemotherapeutic resistance for the SCLC cells.

### Biological pathways differentially altered in small cell lung cancer and non-small cell lung cancer

**Figure 5:**
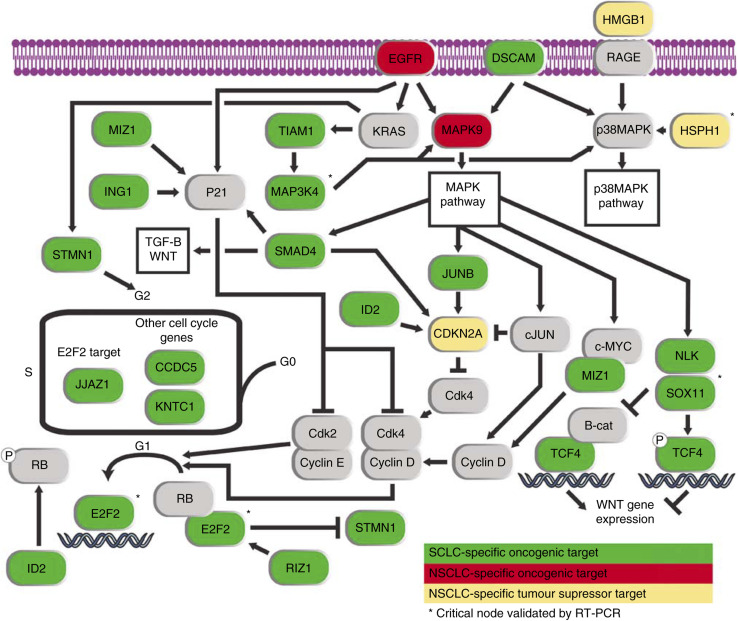
Differential targets of copy number-induced expression changes in key biochemical pathways between SCLC and NSCLC. Strong PSCNA-induced expression differences were identified between SCLC and NSCLC in several key pro-proliferate pathways. Genes with increased expression in SCLC when compared to NSCLC are indicated in green, while genes with increased expression in NSCLC are indicated in red. Genes exhibiting a tumour suppressor like pattern of reduced expression as a result of frequent copy number loss in NSCLC are indicated in yellow. Genes added to the pathways for context but for which no expression differences were detected, are indicated in grey. Critical pathway nodes validated by real-time PCR are indicated with a ^*^.

Further analysis of the differentially expressed genes revealed that a strikingly high number of genes are present in a small set of interconnected pathways. The presence of multiple genes affected by PSCNA in the MAPK and EGFR pathways lead us to examine the known interactors for each of these genes to elucidate a biochemical differentiation between SCLC and NSCLC cells. The results of this analysis are displayed in Figure 5. Twenty-two of the genes differentially altered between SCLC and NSCLC are components of the cell cycle, EGFR, MAPK, p38MAPK, and WNT pathways (Table 1). Four genes (E2F2, SOX11, MAP3K4, and HSPH1), which represent critical nodes in these pathways, were further examined by real-time PCR validating differential expression between SCLC and NSCLC. Pathway information was derived from the Signal Transduction Knowledge Environment (stke.sciencemag.org), the Kyoto Encyclopedia of Genes and Genomes (http://www.genome.jp/kegg), and the following references: Ishitani *et al*, 1999; Lundberg and Weinberg, 1999; Sakamuro and Prendergast, 1999; Taguchi *et al*, 2000; Shaulian and Karin, 2001; Zebedee and Hara, 2001; Hyodo-Miura *et al*, 2002; Schneider *et al*, 2002; Yamagishi *et al*, 2002; Bracken *et al*, 2003; Polager and Ginsberg, 2003; Williams *et al*, 2003; Wu *et al*, 2003; Campos *et al*, 2004; Einarson *et al*, 2004; Li and Guan, 2004; Rubin and Atweh, 2004; Wada and Penninger, 2004; Sasahira *et al*, 2005. Of particular interest was a strong increase in the expression of WNT inhibitors in SCLC cells, namely *NLK*, *SOX11*, and *TCF4*. This remarkable result demonstrates that the WNT pathway may not be a significant player in SCLC.

Additionally we detected a strong difference in the regulatory components of the p38MAPK pathway with the reduced expression of two p38 MAPK activating genes in NSCLC (*HMGB1*, *HSPH1*) and contrasting overexpression of two p38 MAPK activating genes in SCLC (*MAP3K4*, *DSCAM*). We also observed strong PSCNA-related overexpression of several members of the MAPK and cell cycle pathways in both cell types, albeit through different components. In the NSCLC samples, we observed segmental loss and downregulation of the cell cycle inhibitor *CDKN2A* as well as copy number gain and upregulation of *MAPK9* and *EGFR* when compared to SCLC. In contrast, the SCLC cells demonstrate comparatively higher expression of many pro-proliferative genes; these are detailed in Figure 5. Interestingly, several genes with cell cycle inhibitory functions exhibited PSCNA-induced overexpression in SCLC. Owing to likely antagonism of these genes by the many upregulated cell cycle-activating genes, it is possible that they perform a novel role secondary to their primary functions in cell cycle regulation. These differential patterns of oncogenic disruption to cell cycle pathways highlight the need to examine cell type-specific targets for therapeutic pathway intervention. For example, although a recent study has shown that EGFR is expressed at low levels in SCLC, (Tanno *et al*, 2004) our results indicate that the pathway is being activated by overexpression of multiple downstream components, potentially bypassing benefits that may be derived from EGFR-targeted therapy.

## CONCLUSIONS

Whole-genome array CGH in conjunction with global expression profiling analysis has allowed the identification of genes deregulated as a result of PSCNA between SCLC and NSCLC cells. The 159 genes revealed as having strongly divergent expression patterns as a result of copy number alterations identified a remarkable pattern of gene deregulation in several key biological pathways. Cell cycle upregulation in SCLC and NSCLC occurs through drastically different targets, suggesting a need for differential therapeutic target selection. Additionally the WNT pathway, which has recently received much attention for its involvement in NSCLC, appears to be strongly downregulated in SCLC through PSCNA-induced overexpression of inhibitory genes. This work represents the first comprehensive search for the causative genetic alterations distinguishing SCLC and NSCLC by integrating whole-genome expression and copy number analysis platforms.

## Figures and Tables

**Table 1:** Differential deregulation of genes in key biochemical pathways between NSCLC and SCLC

**Gene symbol**	**Gene name**	**Locus**	**Regulation**
*STMN1*	Stathmin 1	1p36.11	SCLC +
*E2F2*	E2F transcription factor 2	1p36.12	SCLC +
*ZNF151 (MIZ1)*	Zinc-finger protein 151 (Myc-interacting zinc-finger protein)	1p36.13	SCLC +
*PRDM2 (RIZ1)*	PR domain-containing protein 3 (Rb protein-binding zinc-finger protein)	1p36.21	SCLC +
*ID2*	Inhibitor of DNA binding 2	2p25.1	SCLC +
*SOX11*	SRY-related HMG-Box gene 11	2p25.2	SCLC +
*MAPK9 (JNK2)*	Mitogen-activated protein kinase 9 (C-JUN kinase 2)	5q35.3	NSCLC +
*MAP3K4*	Mitogen-activated protein kinase kinase kinase 4	6q26	SCLC +
*EGFR*	Epidermal growth factor receptor	7p11.2	NSCLC +
*CDKN2A (p16INK4A)*	Cyclin-dependent kinase inhibitor 2A	9p21.3	NSCLC -
*KNTC1*	Kinetochore-associated protein 1	12q24.31	SCLC +
*HMGB1*	High mobility group Box 1 (Amphoterin)	13q12.3	NSCLC −
*HSPH1*	Heat shock 105 kD	13q12.3	NSCLC −
*ING1 (p33ING1)*	Inhibitor of growth family member 1	13q34	SCLC +
*JJAZ1 (SUZ12)*	Joined to JAZF1 (Suppressor of ZESTE 12)	17q11.2	SCLC +
*NLK*	Nemo-like kinase	17q11.2	SCLC +
*SMAD4*	Mothers against decapentaplegic homolog 4	18q21.1	SCLC +
*CCDC5*	Coiled-coil domain containing 5	18q21.1	SCLC +
*TCF4*	Transcription factor 4	18q21.2	SCLC +
*JUNB*	Oncogene jun-B	19p13.13	SCLC +
*TIAM1*	T-cell lymphoma invasion and metastasis 1	21q22.11	SCLC +
*DSCAM*	Down's syndrome cell adhesion molecule	21q22.2	SCLC +

SCLC=small cell lung cancer; NSCLC=non-small cell lung cancer; +=increased expression in the indicated cell type; −=decreased expression in the indicated cell type.
